# Laminar Airflow vs. Conventional High-Efficiency Particulate Air (HEPA)-Filtered Ventilation in Primary Total Hip and Knee Arthroplasty: A Retrospective Comparative Cohort Study of 1,425 Patients

**DOI:** 10.7759/cureus.116686

**Published:** 2026-09-21

**Authors:** Mahesh Kulkarni, Mohit Nadkarni, Udaya V, Shreesha Parthaje, Hemant Wakankar, Chaitanya Vyawahare

**Affiliations:** 1 Orthopedics, Deenanath Mangeshkar Hospital & Research Center, Pune, IND

**Keywords:** hepa filtration, laminar airflow, operating room ventilation, surgical site infection, total hip arthroplasty, total knee arthroplasty

## Abstract

Background and aim

Laminar airflow (LAF) ventilation is used in orthopedic ORs with the aim of maintaining a controlled surgical environment and reducing the risk of surgical site infections (SSIs). Conventional high-efficiency particulate air (HEPA)-filtered ventilation is also widely used for maintaining air quality in operating theaters. However, the comparative effectiveness of these ventilation systems in primary total hip and knee arthroplasty remains an important clinical consideration. This retrospective cohort study aimed to compare SSI rates among patients undergoing primary total hip and knee arthroplasty in operating theaters equipped with LAF combined with HEPA filtration vs. conventional HEPA-filtered ventilation at a tertiary care hospital.

Materials and methods

This single-center, retrospective comparative cohort study included 1,425 consecutive patients who underwent primary total hip arthroplasty (THA) or total knee arthroplasty (TKA) between January 2022 and December 2024 at a tertiary care hospital. Patients were allocated according to theater ventilation type: Group A, LAF combined with HEPA filtration (n = 695); and Group B, conventional HEPA-filtered ventilation (n = 730). Perioperative protocols, including antibiotic prophylaxis, skin antisepsis, draping, and wound closure, were standardized across groups. Outcomes were assessed using hospital records and a structured telephonic follow-up questionnaire, with SSI classification based on CDC criteria. Multivariable logistic regression was used to adjust for age, sex, BMI, operative duration, diabetes, inflammatory arthritis, renal disease, and procedure type.

Results

The cohort comprised 1,031 TKAs (72.4%) and 394 THAs (27.6%). Baseline characteristics were comparable except for age (marginally higher in the HEPA-only group, p = 0.042) and BMI (higher in the LAF + HEPA group, p < 0.001). Superficial SSI occurred in 28 patients overall (1.96%): 2.16% (15/695) in the LAF + HEPA group vs. 1.78% (13/730) in the HEPA-only group (p = 0.704). No periprosthetic joint infections (PJIs) were identified in either group. On multivariable analysis, ventilation type was not an independent predictor of infection (adjusted OR 0.717; 95% CI 0.334-1.539; p = 0.393), nor were any other assessed covariates.

Conclusions

In this cohort of 1,425 primary hip and knee arthroplasties, no statistically significant association was demonstrated between theater ventilation type and postoperative SSI after adjustment for relevant patient and procedure-related factors. Given the retrospective design, the low number of infection events, and the absence of any PJI in either group, these findings should be interpreted as hypothesis-generating rather than as evidence that ventilation design is clinically unimportant or that perioperative infection-prevention measures are of greater causal importance than ventilation. Larger prospective multicenter studies with adequate power for deep infection outcomes are warranted.

## Introduction

Surgical site infections (SSIs) remain among the most common and clinically significant healthcare-associated infections worldwide, contributing to substantial patient morbidity, prolonged hospitalization, increased healthcare expenditure, and mortality. This concern is particularly relevant to elective total hip arthroplasty (THA) and total knee arthroplasty (TKA), where implanted prosthetic material may serve as a persistent focus for bacterial colonization. Consequently, even a small increase in infection risk can result in serious clinical and economic consequences. The OR environment is considered an important modifiable determinant of SSI risk, with airborne microbial contamination historically recognized as a potential source of infection, especially during clean orthopedic procedures involving prosthetic implants [1,2].

Laminar airflow (LAF) ventilation systems were introduced during the 1960s and 1970s, particularly through the work of Sir John Charnley in orthopedic surgery. LAF provides a continuous, unidirectional flow of high-efficiency particulate air (HEPA)-filtered air over the operative field, with the theoretical aim of reducing airborne particles and microorganisms around the surgical wound. The landmark Medical Research Council (MRC) multicenter trial by Lidwell et al., involving more than 8,000 joint arthroplasties, demonstrated a significantly lower rate of deep infection in ultraclean LAF environments compared with conventional ventilation. These findings contributed to the widespread adoption of LAF as a standard ventilation strategy in orthopedic operating theaters, particularly in Europe and North America [3,4].

However, subsequent evidence has increasingly questioned the presumed protective effect of LAF. Large observational studies and national surveillance analyses have failed to demonstrate a consistent reduction in SSI rates. Brandt et al., using data from the German national nosocomial infection surveillance system (Krankenhaus-Infektions-Surveillance-System) involving nearly 100,000 operations, found no benefit associated with LAF and reported a paradoxically increased risk of severe SSI following hip prosthesis implantation in LAF-equipped theaters. Possible explanations include disruption of LAF by OR personnel, heat generated by surgical lights, and equipment positioning, which may alter airflow and potentially direct contaminated air toward the operative field [5].

Systematic reviews and meta-analyses have similarly challenged the routine use of LAF. A 2017 systematic review in The Lancet Infectious Diseases found no significant reduction in deep SSI following THA, TKA, or abdominal surgery when LAF was compared with conventional HEPA-filtered turbulent ventilation. More recently, a 2023 meta-analysis of 10 randomized controlled trials involving more than one million orthopedic patients reported a pooled OR of 1.70 (95% CI: 1.10-2.64) and a risk ratio of 1.27 (95% CI: 1.02-1.59), suggesting a possible increased SSI risk with LAF. Despite these findings, LAF systems remain widely installed, while recommendations among international guidelines remain inconsistent [6-8].

The present retrospective study therefore compares SSI rates among patients undergoing hip and knee arthroplasty in LAF-equipped vs. conventional HEPA-filtered turbulent-ventilation theaters at a tertiary care hospital from January 2022 to December 2024. By accounting for established confounders, such as age, sex, BMI, operative duration, diabetes mellitus, inflammatory arthritis, renal disease, and type of arthroplasty, this study aims to determine whether ventilation type is independently associated with SSI risk and to provide evidence to inform OR ventilation policy [9,10].

## Materials and methods

Study design

This was a single-center, retrospective comparative cohort study conducted at a tertiary care center, evaluating the association between OR ventilation type and the incidence of SSIs following primary total joint arthroplasty. The study was conducted and reported in accordance with the Strengthening the Reporting of Observational Studies in Epidemiology (STROBE) guidelines for observational studies [11].

Study setting

The study was conducted at a tertiary care center with dedicated orthopedic operating theaters that routinely perform primary total joint arthroplasty. The center has two categories of orthopedic ORs with distinct ventilation configurations. The first comprises theaters equipped with vertical unidirectional LAF ventilation integrated with HEPA filtration (LAF + HEPA group). The second comprises theaters using conventional turbulent mixed ventilation with HEPA filtration alone (HEPA-only group).

Both theater configurations were maintained according to institutional requirements for clean surgical environments, including controlled air exchange and positive pressure relative to adjacent corridors. Air exchange rates were maintained between 15 and 25 air changes per hour in both theater types. Routine environmental monitoring and periodic microbiological air sampling were performed throughout the study period in accordance with institutional OR protocols. The same institutional perioperative infection-prevention practices were applied across both theater categories.

Study period

Medical records of patients undergoing primary total joint arthroplasty, including THA and TKA, between January 2022 and December 2024 were retrospectively reviewed. The three-year study period was selected to provide an adequate sample size and to capture institutional practice during the post-COVID-19 recovery period, when perioperative protocols were standardized across both theater types.

Study population

Patients were eligible if they were aged 18 years or older, underwent primary unilateral or bilateral THA or TKA between January 2022 and December 2024, and had the index procedure performed in either an LAF + HEPA or HEPA-only theater. A minimum follow-up period of 90 days after the index surgery was required. Patients also needed sufficiently complete medical records for data extraction and to be contactable for structured telephonic follow-up.

Patients were excluded if they underwent revision arthroplasty or conversion procedures; had a previous ipsilateral joint infection or periprosthetic joint infection (PJI); had an active systemic infection or sepsis at the time of surgery; or were immunocompromised, including solid-organ transplant recipients receiving immunosuppressive therapy, patients with hematological malignancy, or patients with HIV infection and a CD4 count below 200 cells/µL. Patients with incomplete perioperative records or those lost to follow-up after three documented telephone attempts were also excluded.

Group allocation

Patients were allocated according to the ventilation system of the theater in which the index procedure was performed, as documented in the operative record. Group A comprised 695 patients treated in LAF + HEPA theaters using vertical unidirectional airflow with integrated HEPA filtration and a reported particle-capture efficiency of 99.97% for particles ≥0.3 µm. Group B comprised 730 patients treated in conventional HEPA-only theaters using turbulent mixed ventilation.

Theater allocation was based on scheduled OR availability and was not determined by patient characteristics, surgeon preference, or case complexity. Theater scheduling records were reviewed to assess the potential for selection bias.

Perioperative standardization

To minimize confounding from non-ventilation-related factors, standardized perioperative protocols were applied to both groups throughout the study period. All patients received intravenous cefuroxime 1.5 g within 30 minutes before skin incision. Patients with diabetes mellitus or rheumatoid arthritis received intravenous teicoplanin 400 mg according to the institutional protocol. Surgical-site preparation was performed using 2% chlorhexidine gluconate in 70% isopropyl alcohol [12,13]. Iodine-impregnated adhesive incise drapes were routinely used. All operating personnel wore sterile gowns and double gloves, while body-exhaust suits were used in both theater types.

OR discipline, including personnel numbers and door-opening practices, was governed by institutional policy. Wounds were closed in anatomical layers using standardized techniques, and drains were not routinely used. Low-molecular-weight heparin was administered for thromboprophylaxis according to institutional protocol, beginning 12 hours postoperatively.

Data collection

Data were collected retrospectively from electronic medical records, hospital information systems, operative and anesthesia records, nursing documentation, pharmacy records, microbiology and laboratory databases, outpatient records, and structured telephone questionnaires. A trained data-extraction team used a predefined and piloted proforma.

Baseline variables included age, sex, BMI, American Society of Anesthesiologists physical status, diabetes, hypertension, ischemic heart disease, chronic kidney disease, rheumatoid arthritis, malignancy, steroid use, smoking, and nutritional status where documented. Surgical variables included THA or TKA, laterality, operative duration, surgeon seniority, transfusion requirement, implant type and fixation method, theater ventilation type, and date of surgery. Postoperative variables included hospital stay, wound status at discharge, antibiotic use beyond prophylaxis, culture results, relevant imaging, and readmission. SSI occurrence, classification, and time to diagnosis were recorded according to the CDC National Healthcare Safety Network criteria [14].

Structured telephonic follow-up

Because clinic-based follow-up may miss community-onset SSIs, structured telephonic follow-up was performed by trained research nurses. A pre-piloted questionnaire was administered in the patient’s preferred language and assessed wound redness, swelling, warmth, persistent pain, discharge, unplanned healthcare visits, post-discharge antibiotic prescriptions, wound-related readmissions, diagnosis of wound infection or PJI, and unplanned reoperation, including debridement, irrigation, or revision surgery (Appendix A).

Responses were documented and cross-checked with available clinic, readmission, and microbiology records. Suspected SSIs identified during telephone follow-up were verified through medical records and adjudicated by a blinded senior clinician. Up to three contact attempts were made on separate days and at different times before a patient was considered lost to follow-up.

Primary and secondary outcomes

The primary outcome was the incidence of SSI following index arthroplasty. SSI was classified according to CDC National Healthcare Safety Network criteria as superficial incisional SSI, deep incisional SSI, or organ/space SSI [14]. In this study, organ/space infection included PJI, which was diagnosed according to the 2018 International Consensus Meeting criteria [15,16].

Secondary outcomes included wound-related readmission, return to the operating theater for wound intervention, duration of postoperative antibiotic therapy, and length of the initial hospital stay.

Statistical analysis

Statistical analysis was performed using IBM SPSS Statistics for Windows, version 29.0 (released 2022; IBM Corp., Armonk, NY, USA). Continuous variables were assessed for distribution using the Shapiro-Wilk test and are presented as mean ± SD for normally distributed variables or median with interquartile range for non-normally distributed variables. Categorical variables are presented as frequencies and percentages.

Between-group comparisons were performed using the independent-samples Student’s t-test for normally distributed continuous variables and the Mann-Whitney U test for non-normally distributed continuous variables. Categorical variables were compared using the chi-square test or Fisher’s exact test, as appropriate. The incidence of SSI was compared between the LAF + HEPA and HEPA-only groups.

Multivariable binary logistic regression was performed to evaluate the independent association between theater ventilation type and SSI while adjusting for potential confounding variables, including age, sex, BMI, operative duration, diabetes, inflammatory arthritis, renal disease, and procedure type. Adjusted ORs with 95% CIs were reported. A two-sided p-value <0.05 was considered statistically significant.

## Results

Study population

A total of 1,425 consecutive patients who underwent primary total joint arthroplasty between January 2022 and December 2024 fulfilled the eligibility criteria and were included in the final analysis. Of these, 695 patients (48.8%) underwent surgery in operating theaters equipped with LAF combined with HEPA filtration, whereas 730 patients (51.2%) underwent surgery in conventional HEPA-filtered operating theaters. The overall cohort comprised 1,031 TKAs (72.4%) and 394 THAs (27.6%), with a similar distribution of procedures between the two ventilation groups.

Baseline characteristics

The baseline demographic and perioperative characteristics of the two groups are summarized in Table 1. Overall, the cohorts were well balanced across the principal demographic, comorbidity, and operative variables. Patients in the HEPA-only group were marginally older than those in the LAF + HEPA group (62.5 ± 7.3 vs. 61.7 ± 7.5 years, respectively; p = 0.042). Conversely, BMI was significantly higher among patients in the LAF + HEPA group than in the HEPA-only group (30.6 ± 3.2 vs. 29.8 ± 4.1 kg/m², respectively; p < 0.001).

**Table 1:** Baseline demographic and perioperative characteristics of patients undergoing primary total hip and knee arthroplasty according to OR ventilation type. Continuous variables were compared using Welch’s independent-samples t-test, and categorical variables were compared using the chi-square test with Yates’ correction. HEPA, high-efficiency particulate air; LAF, laminar airflow; THA, total hip arthroplasty; TKA, total knee arthroplasty

Variable	LAF + HEPA (n = 695)	HEPA filter (n = 730)	p-value	Sig.
Surgical procedure				
TKA	500 (71.9%)	531 (72.7%)	0.782	-
THA	195 (28.1%)	199 (27.3%)		
Demographics				
Age (years, mean ± SD)	61.7 ± 7.5	62.5 ± 7.3	0.042	Yes
Gender - Male	300 (43.2%)	310 (42.5%)	0.831	-
Gender - Female	395 (56.8%)	420 (57.5%)		
Comorbidities and risk factors				
BMI (kg/m², mean ± SD)	30.6 ± 3.2	29.8 ± 4.1	<0.001	Yes
Diabetes mellitus	183 (26.3%)	189 (25.9%)	0.897	-
Inflammatory arthritis	81 (11.7%)	81 (11.1%)	0.804	-
Renal disease	29 (4.2%)	27 (3.7%)	0.746	-
Operative details				
Operative time (minutes, mean ± SD)	101.8 ± 28.6	102.6 ± 27.3	0.59	-

No statistically significant differences were observed between the groups with respect to sex distribution, type of arthroplasty, diabetes mellitus, inflammatory arthritis, renal disease, or operative duration. TKA accounted for 71.9% of procedures in the LAF + HEPA group and 72.7% in the HEPA-only group, while THA accounted for 28.1% and 27.3%, respectively (p = 0.782). Similarly, the proportions of male and female patients were comparable between groups. Diabetes mellitus was present in 26.3% of patients in the LAF + HEPA group and 25.9% in the HEPA-only group (p = 0.897), while inflammatory arthritis and renal disease showed similarly balanced distributions. Mean operative duration was also comparable between groups (101.8 ± 28.6 vs. 102.6 ± 27.3 minutes; p = 0.590). These findings indicate substantial baseline comparability between the two cohorts, with statistically significant differences limited to age and BMI.

Postoperative infection outcomes

During the postoperative follow-up period, 28 patients (1.96%) developed a superficial SSI. No confirmed PJIs were identified in either ventilation group following revision of the analytical dataset. As shown in Table 2, superficial SSI occurred in 15 of 695 patients (2.16%) in the LAF + HEPA group and in 13 of 730 patients (1.78%) in the HEPA-only group. The difference in SSI incidence between the two groups was not statistically significant (p = 0.704).

**Table 2:** Comparison of postoperative infection outcomes between patients undergoing primary total joint arthroplasty in operating theaters equipped with LAF + HEPA filtration and those using conventional HEPA-filtered ventilation. Fisher’s exact test was used for all infection outcomes because of the low number of infection events. No confirmed PJIs were identified after revision of the analytical dataset. HEPA, high-efficiency particulate air; LAF, laminar airflow; PJI, periprosthetic joint infection; SSI, surgical site infection

Outcome	LAF + HEPA (n = 695)	HEPA only (n = 730)	p-value
Superficial SSI	15 (2.16%)	13 (1.78%)	0.704
PJI	0 (0.00%)	0 (0.00%)	N/A
Overall infection (SSI + PJI)	15 (2.16%)	13 (1.78%)	0.704

Because of the low number of infection events, Fisher’s exact test was used for the comparison of infection outcomes. No PJI cases were observed in either group, resulting in overall infection rates of 2.16% in the LAF + HEPA group and 1.78% in the HEPA-only group (p = 0.704). Because no PJI events occurred in either group, this study cannot address whether ventilation type influences the risk of deep or organ-space infection. The absence of PJI should not be interpreted as evidence of equivalence between ventilation strategies for that outcome.

Multivariable logistic regression

A multivariable logistic regression analysis was performed to determine whether OR ventilation type was independently associated with postoperative infection after adjustment for potential confounding variables. The model included age, sex, BMI, operative duration, diabetes mellitus, inflammatory arthritis, renal disease, and type of arthroplasty. As shown in Table 3, ventilation type was not independently associated with postoperative infection. Patients undergoing surgery in LAF + HEPA theaters had an adjusted OR of 0.717 compared with those undergoing surgery in HEPA-only theaters (95% CI, 0.334-1.539; p = 0.393).

**Table 3:** Multivariable logistic regression analysis evaluating independent predictors of overall postoperative infection following primary total hip and knee arthroplasty. Continuous variables were standardized per SD. ORs are presented with corresponding 95% CIs. Statistical significance was defined as p < 0.05. Model performance: McFadden’s pseudo-R² = 0.097; Akaike information criterion = 288.3; total sample size = 1,425 patients; overall infection events = 28 (1.96%). HEPA, high-efficiency particulate air; LAF, laminar airflow; THA, total hip arthroplasty; TKA, total knee arthroplasty

Variable	Adjusted OR	95% CI	p-value
Airflow type (HEPA vs. LAF + HEPA)	0.717	0.334-1.539	0.393
Age (per SD increase)	1.358	0.933-1.977	0.111
Female sex	0.999	0.467-2.136	0.997
THA vs. TKA	0.581	0.218-1.545	0.276
BMI (per SD increase)	0.881	0.601-1.290	0.514
Diabetes mellitus	0.67	0.251-1.784	0.423
Inflammatory arthritis	0.642	0.150-2.748	0.551
Renal disease	1.095	0.143-8.374	0.930
Operative time (per SD increase)	0.795	0.545-1.159	0.233

None of the other variables included in the multivariable model demonstrated a statistically significant independent association with postoperative infection. Age, BMI, operative duration, diabetes mellitus, inflammatory arthritis, renal disease, sex, and procedure type all had p-values greater than 0.05. The regression analysis therefore did not identify an independent association between OR ventilation type and postoperative infection within this study population.

Given the low events-per-variable ratio, these adjusted estimates, including the wide CIs around most covariates, should be regarded as exploratory and hypothesis-generating rather than as definitive evidence of the absence of an association.

## Discussion

The principal finding of the present study is that LAF ventilation was not associated with a statistically significant reduction in postoperative SSI compared with conventional HEPA-filtered operating theaters among patients undergoing primary THA and TKA. Among the 1,425 consecutive primary arthroplasty procedures included in the analysis, 28 patients (1.96%) developed a superficial SSI. The incidence was comparable between the LAF + HEPA group and the HEPA-only group, occurring in 15 of 695 patients (2.16%) and 13 of 730 patients (1.78%), respectively. Furthermore, after adjustment for demographic, clinical, and operative variables, OR ventilation type was not independently associated with postoperative infection. These findings indicate that, within the perioperative conditions evaluated in this study, the use of LAF did not result in a measurable reduction in postoperative infection compared with conventional HEPA-filtered ventilation.

The findings also suggest that the contribution of OR airflow configuration to postoperative infection risk may be relatively small when it is considered within a comprehensive perioperative infection-prevention pathway. Modern arthroplasty care incorporates multiple measures designed to minimize infection risk, including appropriate antimicrobial prophylaxis, meticulous skin preparation, sterile draping, controlled OR practices, standardized wound closure, and structured postoperative surveillance. When these measures are consistently implemented, the incremental contribution of a particular ventilation configuration may be difficult to demonstrate, particularly when the baseline incidence of postoperative infection is already low.

Historically, LAF systems were introduced to reduce airborne microbial contamination during orthopedic implant surgery. Early investigations, including the landmark MRC trial led by Lidwell and colleagues, demonstrated reductions in deep infection rates under ultraclean OR conditions and contributed to the adoption of LAF as an important environmental control measure in arthroplasty surgery. However, these investigations were conducted during a period in which perioperative antibiotic prophylaxis, skin antisepsis, surgical draping, implant technology, anesthetic practice, and OR discipline differed substantially from contemporary practice. The infection-prevention environment surrounding modern arthroplasty has subsequently become considerably more comprehensive, making it difficult to attribute changes in infection risk to ventilation alone [3,5].

The present findings are also in keeping with contemporary studies that have evaluated the clinical association between LAF and infection outcomes in arthroplasty. Several observational studies and registry-based analyses have reported no clear reduction in SSI or PJI associated with LAF, while some studies have reported higher infection rates in theaters using unidirectional airflow systems [5-8,10]. The mechanisms underlying these observations remain uncertain. Although unidirectional airflow is designed to reduce the concentration of airborne contaminants in the operative field, its effectiveness under actual surgical conditions may be influenced by factors such as surgical lighting, heat generated by personnel and equipment, movement within the OR, positioning of equipment, and disruption of airflow patterns. These factors may reduce the extent to which the theoretical environmental advantages of LAF translate into clinically meaningful reductions in postoperative infection [5-8].

Evidence from contemporary large-scale arthroplasty cohorts further supports the uncertainty surrounding the additional clinical benefit of LAF. A nationwide Danish study involving 92,152 primary THA procedures found no significant difference in the hazard of revision for PJI between LAF and turbulent airflow after adjustment for patient- and surgery-related factors (HR, 0.99; 95% CI, 0.78-1.26) [10]. Although differences in study design, outcome definitions, follow-up duration, and institutional practices should be considered when comparing these findings with the present study, the overall evidence suggests that the relationship between ventilation configuration and clinically apparent infection is complex and may be less substantial than historically anticipated.

An important strength of the present study is the relatively large cohort of 1,425 consecutive primary arthroplasties managed within a single institution under standardized perioperative protocols. Both ventilation groups were exposed to the same institutional approaches to antimicrobial prophylaxis, skin preparation, surgical draping, wound closure, and postoperative care. This consistency reduced variation in several important infection-related factors and allowed the comparison to focus more directly on the difference in OR ventilation. In addition, postoperative outcome assessment incorporated hospital records and structured telephonic follow-up, providing an opportunity to identify wound complications that may have been managed outside routine postoperative clinic visits. Suspected infections identified during telephone follow-up were subsequently verified against available clinical and microbiological records, further strengthening outcome ascertainment.

Another important observation was the absence of confirmed PJI in either study group following revision of the analytical dataset. All 28 recorded infection events were classified as superficial SSI, resulting in an overall infection incidence of 1.96%. The absence of deep or organ-space infections limits the ability to determine whether ventilation type has any association with the more severe forms of arthroplasty infection. Nevertheless, the low overall infection burden observed in both groups provides evidence that low postoperative infection rates were observed under both ventilation configurations when combined with the standardized perioperative practices used at the study center.

The statistically significant baseline differences in age and BMI between the groups also warrant consideration. Patients in the HEPA-only group were marginally older, whereas patients in the LAF + HEPA group had a higher mean BMI. However, neither age nor BMI was independently associated with postoperative infection in the multivariable analysis. Similarly, diabetes mellitus, operative duration, renal disease, inflammatory arthritis, sex, and procedure type were not independently associated with infection. These findings suggest that the observed similarity in infection rates was not readily explained by differences in the measured demographic or clinical characteristics. Nevertheless, the absence of statistical associations should not be interpreted as evidence that these factors have no biological or clinical relevance to infection risk, particularly given the small number of infection events in the present cohort.

The findings have potentially important implications for OR infrastructure and healthcare resource allocation. LAF systems require dedicated engineering infrastructure, installation and maintenance costs, periodic technical validation, and appropriate environmental control. Conventional HEPA-filtered ventilation may provide an alternative approach to maintaining controlled OR air quality without the additional infrastructure associated with LAF. In the context of the present findings, institutions considering new arthroplasty OR construction or renovation may therefore need to weigh the potential environmental advantages of LAF against its additional infrastructure and maintenance requirements. Such decisions should, however, incorporate local infection rates, existing ventilation infrastructure, institutional standards, and economic considerations rather than relying on the findings of a single-center observational study.

From a clinical perspective, the results support a multifaceted approach to infection prevention in contemporary arthroplasty. Patient optimization, appropriate antimicrobial prophylaxis, meticulous skin antisepsis, adherence to sterile surgical practices, appropriate OR discipline, standardized wound management, and timely recognition of postoperative wound complications remain important components of infection prevention [11-14]. Ventilation should therefore be considered as one component of the broader OR environment rather than as an isolated determinant of postoperative infection. The comparable infection rates observed in the present study suggest that achieving consistently low infection rates may depend more strongly on the coordinated implementation of multiple perioperative measures than on the choice between the two ventilation configurations evaluated.

Strengths

The present study has several strengths that enhance the relevance of its findings. It included a relatively large cohort of 1,425 consecutive primary arthroplasty procedures and directly compared two ventilation configurations within the same institutional environment. The use of standardized perioperative protocols reduced heterogeneity in several important infection-prevention measures between the groups. In addition, combining hospital-based outcome data with structured postoperative telephone follow-up increased the opportunity to identify wound complications that may not have been documented during routine institutional follow-up. Verification of suspected infections against available clinical records and microbiological information further supported the accuracy of outcome classification. Finally, multivariable logistic regression was used to account for several measured demographic, clinical, and operative factors that could potentially influence postoperative infection risk.

Limitations

Several limitations should be considered when interpreting these findings. The retrospective, observational design introduces the possibility of residual confounding despite adjustment for selected demographic, clinical, and operative variables. Although theater allocation was based on scheduled OR availability rather than documented patient characteristics or surgeon preference, unmeasured differences between cases performed in the two theater types cannot be completely excluded. The 28 infection events relative to the nine predictors included in the regression model yielded an events-per-variable ratio of approximately 3, well below conventional recommendations of ≥10. Therefore, adjusted estimates, including the wide CIs, should be interpreted as exploratory rather than definitive, and there is a meaningful risk of model overfitting or instability. The very low number of infection events also limits the statistical power of the study. No PJI occurred in either group, precluding any meaningful assessment of the relationship between ventilation and PJI, which is an important clinical outcome in arthroplasty. Accordingly, the absence of a statistically significant association should not be interpreted as definitive evidence of equivalence between ventilation systems.

Environmental measurements were another limitation. Detailed data regarding OR traffic, frequency of door opening, real-time airborne particle concentrations, airflow velocity, and intraoperative microbiological conditions were not available for the present analysis. Consequently, the study evaluated the clinical outcomes associated with the overall ventilation configurations rather than determining the specific environmental mechanisms through which either system might influence contamination. Furthermore, no confirmed PJI cases were identified, preventing meaningful assessment of the relationship between ventilation type and deep or organ-space infection. Finally, because the study was conducted at a single tertiary care center with standardized institutional protocols, the findings may not be directly generalizable to hospitals with different patient populations, perioperative practices, infection-prevention protocols, or OR ventilation systems.

Future research should include multicenter prospective studies with larger sample sizes and sufficient numbers of infection events to provide more reliable estimates of the association between ventilation type and both superficial SSI and PJI. Such studies would benefit from concurrent environmental monitoring, including airborne particle measurements, airflow characterization, microbiological air sampling, OR traffic assessment, and documentation of door-opening frequency. Incorporating cost-effectiveness analyses would also help determine whether any incremental clinical benefit associated with LAF, if present, justifies its additional installation and maintenance requirements. These data would provide a more comprehensive basis for determining the role of OR ventilation in contemporary arthroplasty infection prevention.

## Conclusions

In this retrospective comparative cohort study of 1,425 primary total hip and knee arthroplasties, no statistically significant association was demonstrated between theater ventilation type and postoperative SSI, either before or after adjustment for measured patient and operative characteristics (adjusted OR 0.717; 95% CI 0.334-1.539; p = 0.393). Given the observational design, the low number of infection events, the resulting limitations on model precision, and the absence of any PJI events in either group, these findings should be regarded as hypothesis-generating. The present study does not establish the relative contribution of perioperative infection-prevention practices and ventilation design to postoperative infection risk; rather, it demonstrates only that ventilation type was not shown to be an independent predictor of infection in this cohort. Adequately powered, ideally prospective, multicenter studies capable of evaluating deep infection outcomes are needed before conclusions can be drawn about the comparative value of LAF vs. conventional HEPA-filtered ventilation.

## Figures and Tables

**Figure 1:**
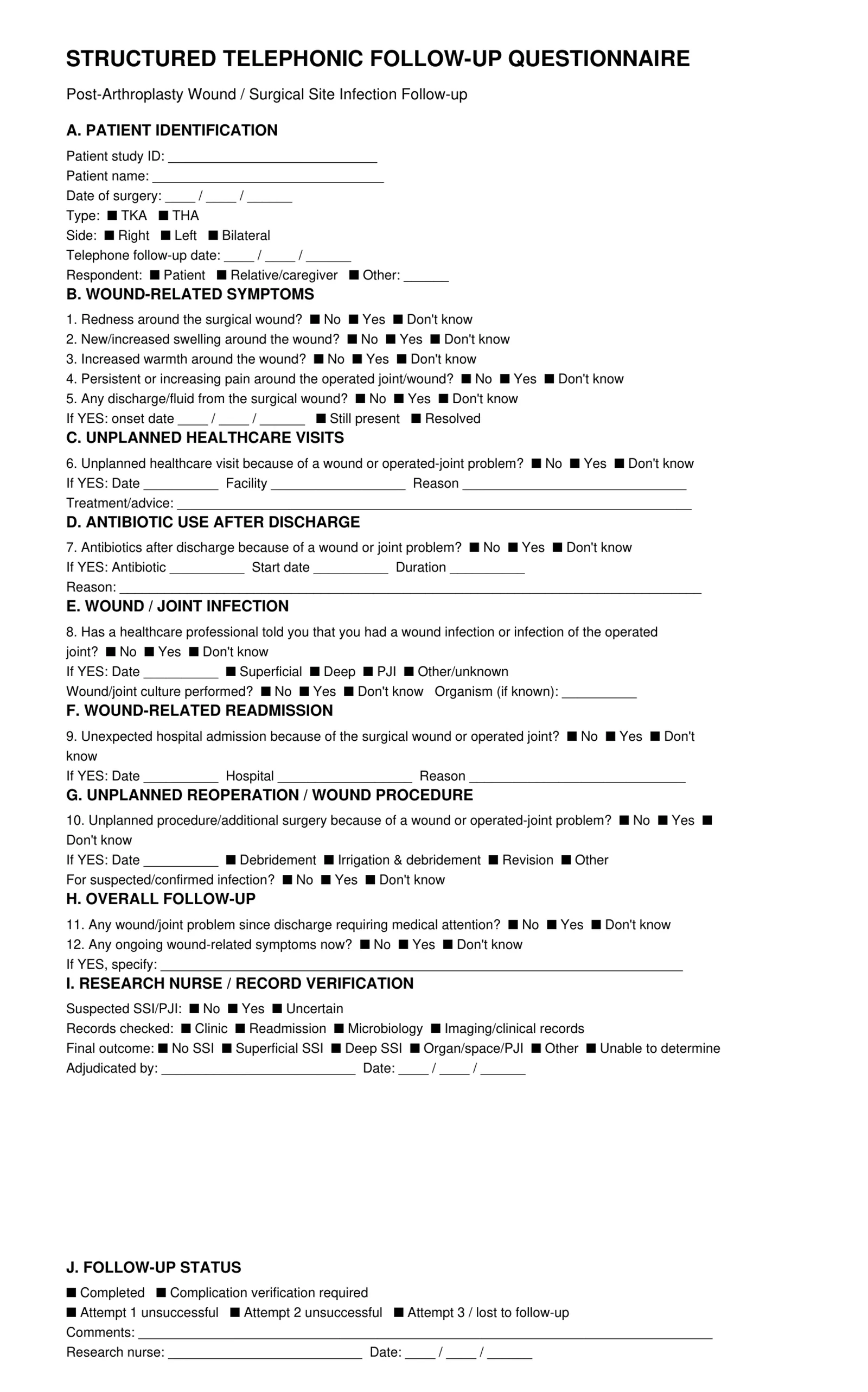
Study questionnaire.

## Appendices

Appendix A
